# Optical Fiber Sensors and Sensing Networks: Overview of the Main Principles and Applications

**DOI:** 10.3390/s22197554

**Published:** 2022-10-05

**Authors:** Cristiano Pendão, Ivo Silva

**Affiliations:** ALGORITMI Research Centre, University of Minho, 4800-058 Guimarães, Portugal

**Keywords:** optical fiber sensors, optical fiber sensing, optical fiber sensing networks, sensor networks, wireless sensor networks

## Abstract

Optical fiber sensors present several advantages in relation to other types of sensors. These advantages are essentially related to the optical fiber properties, i.e., small, lightweight, resistant to high temperatures and pressure, electromagnetically passive, among others. Sensing is achieved by exploring the properties of light to obtain measurements of parameters, such as temperature, strain, or angular velocity. In addition, optical fiber sensors can be used to form an Optical Fiber Sensing Network (OFSN) allowing manufacturers to create versatile monitoring solutions with several applications, e.g., periodic monitoring along extensive distances (kilometers), in extreme or hazardous environments, inside structures and engines, in clothes, and for health monitoring and assistance. Most of the literature available on this subject focuses on a specific field of optical sensing applications and details their principles of operation. This paper presents a more broad overview, providing the reader with a literature review that describes the main principles of optical sensing and highlights the versatility, advantages, and different real-world applications of optical sensing. Moreover, it includes an overview and discussion of a less common architecture, where optical sensing and Wireless Sensor Networks (WSNs) are integrated to harness the benefits of both worlds.

## 1. Introduction

The development of optical fiber technology marked an important step in global communications technology. In the 70s, the emergence of optical fibers with low attenuation [1] enabled long-distance communications with high bandwidth. Since these advancements, the production volume has continued to grow, and by 2000, optical fibers had already been rapidly installed around the world [2].

The evolution of optical fiber technology also enabled the development of devices for optical processing entirely in fiber, reducing insertion losses and improving the quality of processing [3]. One factor that contributed to the full migration of optical fiber technology was the identification of photosensitive optical fibers. This discovery was made in 1978 by Hill et al. [4] and led to the development of the optical Fiber Bragg Grating (FBG). In parallel with the interest and use in optical communications, the Bragg gratings have gained a prominent position in optical fiber sensors, due to their versatility in different sensing applications [5,6]. Several markets, such as aeronautics [7], aerospace [8], civil engineering [9,10,11,12,13,14,15], and biological [16] or environmental monitoring [17], have assimilated the advantages of this technology.

Optical fibers provide sensing solutions for many types of applications and environments with high performance. The design of the fiber sensors can take advantage of one or several optical parameters of the guided light, such as intensity, phase, polarization, and wavelength. The optical fiber offers dual functionality: measurement of several parameters through changes in the properties of light propagating through the fiber; and functions as a communications channel, therefore dispensing an additional dedicated communication channel, and thus presenting an advantage concerning all other sensing technologies.

Optical fiber sensors are electromagnetically passive. This characteristic is very important as it allows the use of optical sensors where other types of sensors cannot be employed, for example, in high and variable electric field environments where there are explosion risks. Furthermore, the silica compound, which is the basic transduction material of optical fiber, is resistant to most chemical and biological agents and thus can be used in this type of environment and materials. Another advantage is that optical fiber sensors can be small and lightweight [18].

The fiber has low optical attenuation enabling propagation over long distances (kilometers) between monitoring stations. The low attenuation is also important to perform multiplexed measurements. By using a single optical source and detection unit, it is possible to operate large arrays of distributed sensors without active optoelectronic components in the measurement area. In turn, the electromagnetic passiveness and environmental resistance can be maintained [19,20].

Optical fiber sensor systems are normally used in pre-defined positions. Therefore, extensive lengths of fiber optic cable are necessary for connecting all the sensors and creating an optical fiber network, which can be expensive and impractical. In recent years, Wireless Sensor Networks (WSNs) have gained considerable attention for their effectiveness in acquiring information on parameters such as temperature, pressure, acceleration, or vibration [21]. Nevertheless, most WSN systems do not integrate optical fiber sensors and do not benefit from their special characteristics and advantages. Therefore, the integration of optical fiber sensors in WSNs introduces benefits and new capabilities for the design of advanced hybrid-sensing systems [22].

**Figure 1:**
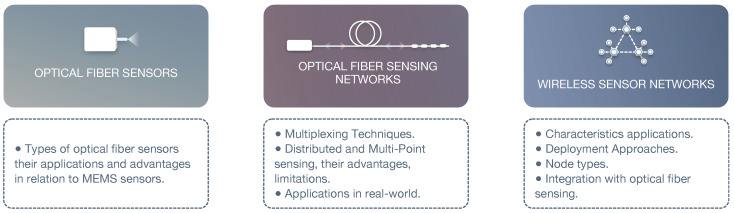
Content Outline.

This paper provides a review of optical fiber sensors, in addition to optical fiber sensing networks and their real-world applications. Moreover, we analyze the integration of optical fiber sensing with OFSNs, allowing us to combine the benefits of both areas. This integrated approach is relatively recent but can significantly evolve and gain attention in the future. Figure 1 provides an outline of the main topics addressed in this paper.

The document is organized as follows: Section 2 presents the different types of optical fiber sensors, their main operating principles, and applications. Furthermore, the main characteristics of these sensors are summarized and compared; Section 3 describes the fundamentals of optical fiber sensing networks (multi-point and distributed), how they are arranged, their main areas of application and the characteristics of two commercial solutions are compared; Section 4 addresses the relevance of optical sensing integration in WSNs; Finally, the conclusions and future prospects are provided in Section 5, reflecting the most important aspects of this paper.

## 2. Optical Fiber Sensors

An optical fiber is a cylindrical dielectric waveguide, where both the core and the cladding are composed of glass or plastic, and the surrounding coatings used to protect the optical fiber are made of acrylate or polyimide materials. Optical fibers can be multi-mode or single-mode. An optical fiber sensor expands or contracts according to strains or temperature variations. When light is sent down the fiber to the sensor, it is modulated according to the amount of expansion or contraction. Subsequently, the sensor reflects back an optical signal to an analytical device, which translates the reflected light into numerical measurements of the change in the sensor length. These measurements indicate the level of strain or the temperature [11].

As mentioned before, optical fiber sensors have several advantages relative to other sensor technologies for a variety of applications with extensive potential in sensing applications. Some advantages of optical fibers with regard to sensing include their small size, no requirement of electrical power at the remote location, and many sensors can be multiplexed along the length of the fiber by using light wavelength shift for each sensor, or by sensing the time delay as light passes along the fiber through each sensor [20]. Because optical fiber sensors are immune to electromagnetic interference and do not conduct electricity, they can be used in hazardous environments where high-voltage electricity or flammable material such as jet fuel may be present. Optical fiber sensors can also be designed to resist high temperatures [12].

For these reasons, the application environments range from dangerous scenarios where there are radioactive, chemical, and other industrial-based hazards to more common and simple uses. However, the development of certain types of optical fiber sensors, for example, in corrosion detection, remain in its infancy [11,23].

**Figure 2:**
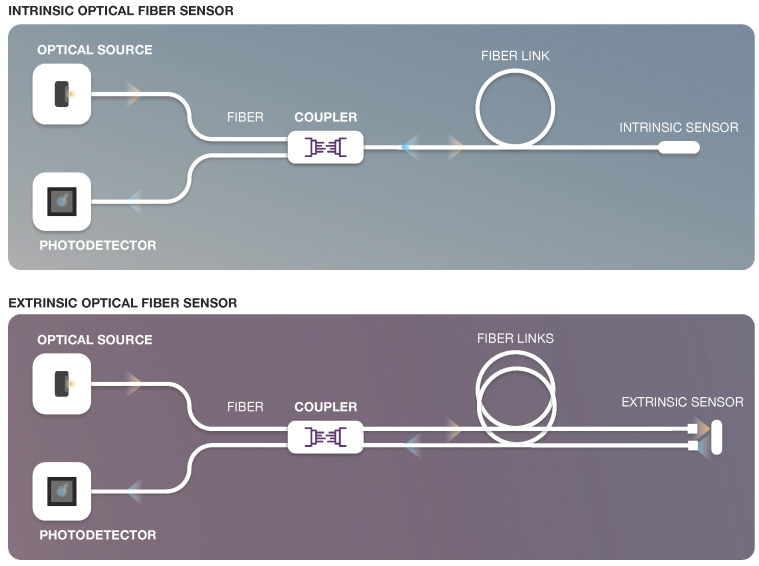
Types of optical sensors considering the location of the sensor.

In the literature, optical fiber sensors can be classified or categorized considering different aspects. These sensors are frequently grouped according to the sensor location in the fiber, and the operating principle or the application [24,25,26,27]. When considering matters of application, optical sensors can be categorized considering the type of parameters they are intended to measure, namely: physical (e.g., strain, temperature), chemical (e.g., oil parameters, pH, ammonia, detergents, pesticides and humidity) [28,29] or bio-medical (e.g., oxygen, carbon dioxide, proteins, cells, proteins, and DNA) [29,30,31,32,33]. Concerning the sensor location, the optical sensor can be classified as intrinsic or extrinsic [22] (Figure 2).

Intrinsic sensors (upper part of Figure 2) directly use an optical fiber as the sensitive material (sensor head) and also as the medium to transport the optical signal with the information measured. They operate via direct modulation of the light guided into the optical fiber, and the light does not leave the fiber, except at the detection end. In this type of sensor, physical perturbations modify the characteristics of the optical fiber, changing the properties of the light carried by the fiber. Alternatively, the modulated light may be coupled back into the same fiber by reflection or scattering and then guided back to the detection system. The simplest fiber sensors vary the intensity of the light and require only a light source and a detector.

Intrinsic optical fiber sensors can be used in distributed sensing over large distances to measure different parameters, for example: temperature can be measured by analyzing the Raman scattering of the optical fiber or by using a fiber bearing an evanescent loss that varies with temperature; electrical voltage can be measured by analyzing the polarization of light as a function of voltage or electric field considering the nonlinear optical effects in specially doped fibers; angles can be measured through the Sagnac effect; and, direction recognition is possible using special long-period fiber grating [18].

Furthermore, intrinsic optical fiber sensors are used in different fields for different purposes. They are employeed as hydrophones for seismic and sonar applications [34,35,36,37,38,39], building a system with several sensors per fiber cable. An advantage of these sensors is that they can simultaneously measure the temperature and acoustic pressure at the same location [40]. This is particularly useful when acquiring information from small complex structures. In oil wells, intrinsic optical fiber sensors are used for measuring temperature and pressure with precision.Simultaneous temperature and strain sensing over large distances is also possible exploring the Brillouin scattering effects, which enable sensing over larger distances (>30 km) [18]. Another application of these sensors is in healthcare for medical imaging and diagnosis [41,42], due to their higher resistance to chemical agents and immunity to electromagnetic interference. Intrinsic sensors are also used in airplanes [43] and cars [44] as high-accuracy optical fiber gyroscopes for navigation purposes.

An extrinsic or hybrid optical fiber sensor (usually based on a multimode fiber cable) (see Figure 2) guides the light to/from a location where the optical sensor head is located. The sensor head is external to the optical fiber and is based on miniature components that are used to modulate the properties of light in response to environmental changes associated with physical perturbations of interest. The optical energy is transmitted to the head of the sensors from one end of the fiber, and the other end of the fiber is modulated and coupled to the optical sensor.

In this type of sensor, the optical fiber is simply used to guide the light to and from a location where an optical sensor head is located. The typical configuration is one fiber to transmit to the sensor head, and a second fiber to guide the modulated light back to the optical detector. Another configuration may use only one fiber, the modulated light may be coupled back by reflection or scattering and then guided back to the detection system. This type of sensor can reach places that other transmission methods cannot, for example, inside an aircraft jet engine for temperature measurement, or in locations with extreme electromagnetic fields. Extrinsic sensors are used to measure vibration, rotation, displacement, velocity, acceleration, torque, and temperature [18]. Extrinsic optical fiber sensors provide resistance to noise and signal corruption, however, integration with other types of sensors can be difficult since other sensors produce an electrical output that then has to be converted into an optical signal. Fabry–Perot interferometers are an example of extrinsic sensors, which have a cavity at the end of the fiber where light comes out from and allows users to perform sensing of numerous parameters, e.g., pressure and temperature in geothermal wells [45], ultrasound, humidity, liquid level sensing [46], and structural health monitoring of bridges [12].

**Figure 3:**
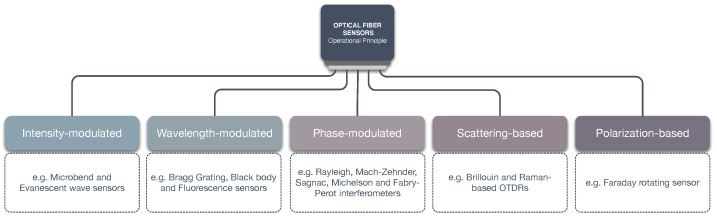
Types of optical fiber sensors with respect to their principle of operation.

With regard to the principle of operation, the optical fiber sensors can be classified as intensity-modulated, wavelength-modulated, phase-modulated, scattering-based or polarization-based (Figure 3) [24,25,26,27]:

- Intensity-modulated sensors were among the first optical fiber sensors to be developed [26]. These sensors can detect physical changes or perturbations in the received light (bend loss, attenuation, evanescent fields). Simplicity and lower costs present the advantages of this optical sensor type; however, these sensors are susceptible to fluctuations in optical power loss leading to false readings, and therefore requiring a reference system to minimize the problem.
- Wavelength-modulated sensors measure the wavelength change in the fiber. Examples of wavelength-modulated sensors include black body sensors, fluorescence sensors, and the Bragg grating wavelength-modulated sensors. The FBG sensor represents the most popular type of wavelength-modulated sensor and is frequently used in different applications since it is capable of single-point or multi-point sensing.
- Phase-modulated sensors use the interferometry principle to measure interference of the optical fiber light. These sensors are popular owing to their high sensitivity and accuracy; however, this also translates to a higher cost. The most popular phase-modulated sensors include the Mach–Zehnder, Sagnac, Michelson, and Fabry–Perot interferometers.
- Scattering-based sensors use an Optical Time Domain Reflectometer (OTDR) to detect changes in the scattered light. These sensors are very popular since they enable distributed sensing along the length of the fiber with interesting applications in structural health monitoring, and measuring changes in strain.
- Polarization-based sensors detect changes in the light caused by an alteration in the polarization state. These sensors exploit the birefringence phenomenon in the optical fiber, where depending on the polarization the refractive index changes. When applying strain to the optical fiber, the birefringence effect occurs and results in a detectable phase difference.

In the literature, the most popular optical fiber sensors are classified into three main groups: Grating-Based, Interferometric and Distributed [47]. Next, we will provide more details about the sensors in each of these three categories, with more focus on those with higher potential for sensing networks.

### 2.1. Fiber-Bragg Grating Sensors

FBGs [4] are simple, versatile, and small intrinsic sensing elements that have all the advantages generally attributed to optical fiber sensors. Since the information to be measured is encoded in the wavelength of the structure, which is an absolute parameter, FBG sensors can be easily multiplexed in multi-point sensing networks.

**Figure 4:**
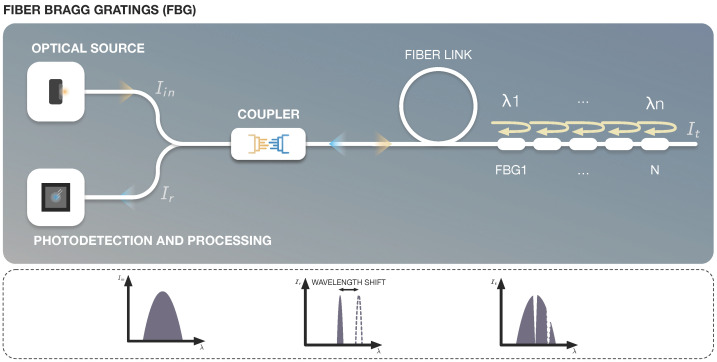
Fiber Bragg Gratings.

The FBG sensor works by exposing a section of the optical fiber core to a periodic pattern of UV light, which results in a permanent alteration of the refractive index of the core. This process provides spectrally controlled reflective properties to the UV-light-treated portion of the fiber [4]. The reflected wavelength exhibits high sensitivity to extension and temperature variations as shown in the lower part of Figure 4. These sensors are capable of eliminating the problems of amplitude or intensity variations because they are integrated into the light guiding core of the fiber and are wavelength encoded.

The fabrication process of FBGs has undergone continual improvement in recent decades to enable functional FBG operation in harsh environments with very high temperatures, such as the oil and gas industries, aircraft engines, among others. Recently, femtosecond laser technology was explored for fabricating microstructures, including FBGs [48]. This fabrication process enabled FBG sensors to operate at very high temperatures, for example, a type-II FBG fabricated on a conventional single-mode fiber achieved long-term thermal stability at 1000 °C for several hundred hours [49], and an FBG based on pure-silica Photonic Crystal Fiber (PCF) was able to operate at ∼1300 °C [50]. PCF is a type of fiber that contains air holes along the fiber in the cladding and/or core. A detailed description of femtosecond laser technology and several exploratory works can be found in [48].

The Bragg wavelength $\lambda_{B}$ in FBG sensors or the wavelength of the reflected light is given by [4]:

(1) $$\lambda_{B}=2n_{eff}\Lambda$$

where $n_{\mathrm{eff}}$ is the effective refractive index of the fiber core and Λ is the grating period. The Bragg wavelength varies with changes in the grating period and effective refractive index, as can be seen in (1). The grating period is affected by variations in the strain, and the effective refractive index is affected by variations in the temperature.

In order to measure each physical parameter in FBGs, the temperature and strain effects need to be separated from each other. The use of a reference grating presents a practical and simple approach to separate the effects caused by temperature and strain. FBG sensors require demodulators, also known as interrogators, that are used to extract measurand information from the light signals coming from the sensor heads. Since the information is encoded in the Bragg wavelength, the interrogators are expected to read the shifts in the Bragg wavelength and provide the measurand data. Figure 4 presents the interrogator of an FBG sensor.

Typical strain FBG responses include ∼0.64, ∼1, and ∼1.2 pm/μϵ (μϵ = micro-strain) for the Bragg wavelengths of around 830, 1300, and 1550 nm, respectively, [51]. Despite being dependent on the FBG type, the temperature response is typically 6.8, 10, and 13 pm/°C, respectively, [52]. FBG sensors are used in various areas to measure numerous physical parameters [53], such as: temperature and strain [54,55], salinity of sea water [56], pressure and temperature of geothermal wells [45], and displacement and liquid levels [53].

### 2.2. Interferometric Sensors

**Figure 5:**
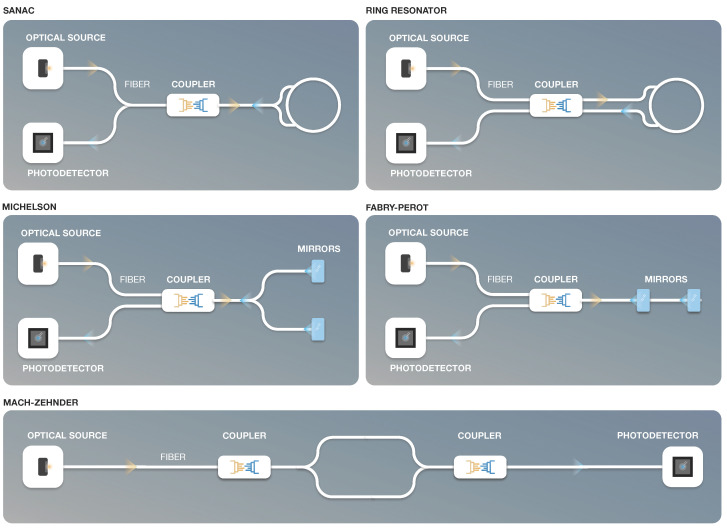
Fiber optic interferometer types.

Interferometric optical fiber sensors, also referred to as interferometer sensors, are phase-modulated sensors that measure the interference of the optical fiber light. The most popular types of interferometers include the: Mach–Zehnder, Sagnac, Michelson, Fabry–Perot and ring resonator. Figure 5 illustrates a schematic representation of different fiber optic interferometers.

The Mach–Zehnder interferometer [57,58] works by sending a light beam split into two parts so that the signal propagates through a reference arm and a sensing arm. It then measures the phase shift between the two light beams which are recombined at the detector. The phase shift occurs due to a change in sensing arm length following mechanical or thermal strain. This type of interferometer is mostly used to test telecommunication industry networks, for underwater sensing as a hydrophone [59], for sensing temperature and refractive index [60], in the healthcare industry for optoacoustic imaging applications [61], and as a heart rate and respiratory rate sensor [62].

The Michelson interferometer [63] is similar to the Mach–Zehnder, but instead of having a second beamsplitter, it uses mirrors to reflect the light in the reference and sensing arms back to the source. Some of its applications are as a refractive index sensor [64] and as a hydrophone sensor [65].

The Fabry–Perot interferometer [66] is an extrinsic sensor that uses two parallel reflective surfaces separated by a certain distance and measures the interference between the transmitted and received signal. It is widely used to monitor structural components and downhole pressure in the oil and gas industry [45,67], and for sensing temperature, acoustic waves, ultrasound waves, gas, and liquid levels, among others [46].

The ring resonator [68] detects inertial rotation normal to the plane of the ring resonator and may be used for optical switching and photonic biosensors, among other applications.

The Sagnac interferometer [69] is used to measure the phase shift between two different wavelengths propagating in opposite directions. An interesting type of Sagnac interferometer is the fiber optic gyroscope, initially introduced in 1976 [70]. It detects the phase shift in the optical fiber, assuming a rotating fiber optic coil with two light waves traveling in opposite directions in the coil. These light waves will travel different distances, which results in different travel times and different phases between the two waves. The phase difference ΔΦ is given by:

(2) $$\Delta \Phi =\frac{8\pi N}{\lambda c}A\times \Omega$$

where *N* is the number of coil turns, λ is the wavelength in a vacuum, *c* is the speed of light, *A* is the area vector of the fiber coil, and Ω is the rotating rate (angular frequency) vector.

The output response of a gyroscope sensor is a raised cosine function with respect to the phase difference. When a point corresponds to ΔΦ=0 in the response curve, it is at maxima (or minima), and therefore the sensitivity is low and the rotational direction of the fiber coil cannot be distinguished due to the symmetric response. Thus, the operating point has to be shifted to a position where the response is not zero. This is achieved using a phase modulator at the end of the fiber coil.

The fiber coil rotates in the clockwise (CW) direction. In order for the rotating light waves (one in the CW direction and another in the counter-clockwise (CCW) direction) to meet and interfere with each other at the same exit point, they should have entered the coil at different time instants. Since the coil is rotating, different entering times mean different entering points.

When compared with other alternatives, the fiber optic gyroscope has an important advantage—ruggedness. Since it does not contain any moving parts, in contrast to mechanical and ring laser gyroscopes, it overcomes cross-axis vibration and acceleration achieving higher resolution than ring laser gyroscopes. The performance of these sensors has improved significantly rendering them suitable for meeting the most demanding of requirements in gyroscope accuracy, finding applications in military and commercial markets [71,72]. With the evolution of technology, the price of these sensors continues to decrease due to the expansion of the optical fiber communications market and reduced costs of the building components [73]. Hence, they are considered the most cost-effective solution for high-accuracy inertial navigation applications [74], being particularly useful in GNSS-denied environments where it is necessary to locate a vehicle recurring to inertial navigation.

Optical fiber acoustic sensors or optical fiber hydrophones use interferometry for measurements in underwater environments [34,38,39]. These sensors are often preferred as substitutes for piezoelectric ceramic sensors owing to the advantages associated with optical fibers, namely, high sensitivity, high dynamic range, and immunity to electromagnetic interference. Fiber optic hydrophones rely on the operating principle of interferometers and may be based on Mach–Zehnder, Sagnac, Michelson, or Fabry–Perot interferometers to detect the phase change of light which is usually introduced by pressure. Propagating acoustic waves cause pressure-induced variation in the refractive index, which in turn produces a phase shift in the light propagating through the fiber [75]. There are several applications of fiber optic hydrophones, for instance, in seismic exploration for oil reserves [76] and geoacoustic seafloor exploration [77].

The work in [78] reviews several optical fiber-based sensors for real-time measuring and monitoring of volatile organic compounds (VOCs), such as alcohols, carbonyls, alkanes, among others. This paper includes several applications of interferometers for sensing volatile organic compounds, for example, a sensor using a polydimethylsiloxane (PDMS) sensing film over a glass substrate. PDMS has unique features that cause it to change its refractive index when it interacts with various VOCs causing a fringe shift in the interferometer.

### 2.3. Distributed Sensors

**Figure 6:**
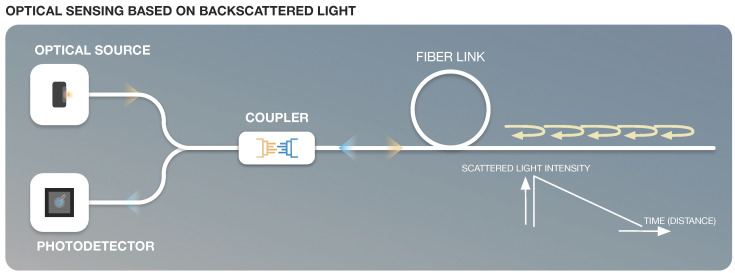
Optical Sensing based on backscattered light.

**Figure 7:**
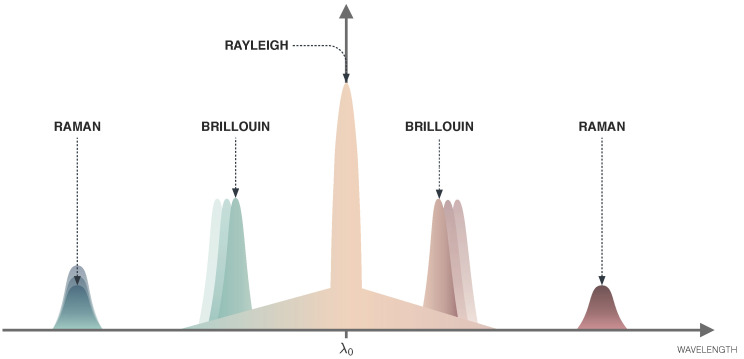
Spectra of scattered light in optical fiber.

Optical Time Domain Reflectometers (OTDRs) are some of the most well-developed in-line sensors based on the scattered light propagating through the fiber, which contains Rayleigh, Brillouin and Raman scatterings as displayed in Figure 6 and Figure 7. In addition to the original wavelength (called the Rayleigh component), the scattered light contains components at wavelengths that are higher and lower than the original signal (known as the Raman and Brillouin components). These shifted components contain information about the local properties of the fiber, such as strain and temperature.

The functioning of OTDR [79] details a process whereby light injected into the system is shortly pulsed in order to achieve spatial resolution. As the backscattered light is detected with a certain delay relative to the emitted beam, the region where the scatter originated is identified. Hence, the loss in this region can be measured because the intensity of the scattered light in that region is different. Using the frequency or arrival time of the scattered light allows determination of the measurand amplitude and location.

Rayleigh scattering results from the interaction of light with refractive index fluctuations in the fiber core, that appear in spatial scales much shorter than the light wavelength. Rayleigh scattering has the same light frequency as the incident light and is very weak, especially in single-mode fibers [80], and thus is not frequently used in sensing applications.

The Brillouin scattering appears from the interaction of light with acoustic modes in the medium, which are induced by the light propagation [80]. Determining the Brillouin frequency shift relative to the incident light provides a measure of temperature or strain, and allows distributed sensing in long-range fibers. This frequency shift is an intrinsic property of any silica fiber, and therefore allows the production of low-cost sensing elements. The measurements are stable over time because the optical effect only depends on the fiber material. Therefore, Brillouin scattering has been used in distributed sensing of large structures, mainly sensing strain in oil wells, pipelines, bridges, or power lines [18,81]. Brillouin-OTDR technology can be used to measure strain and temperature continuously at any point distributed along an optical fiber, thus it is used for full-scale monitoring of large structures such as tunnels, dams, pipes, subways, and large bridges in the order of hundreds of kilometers, where point-measurement monitoring techniques cannot be used. In addition, the optical fiber in Brillouin-OTDR functions both as a sensor and as a transmission medium, enabling long-distance and real-time remote monitoring [11].

Distributed strain sensing with Brillouin scattering achieves a range up to 30 km with a 20 μϵ (micro-strain) resolution (more advanced schemes can achieve higher resolutions, reaching up to 0.1 μϵ) [27]. The sensing point associated with a physical perturbation can be resolved up to 1 m over a 10 km length; however, the accuracy decreases as distance increases. Brillouin scattering for temperature sensing has a resolution of 0.5 °C [27].

Raman scattering results from the interaction of the propagating light with molecular vibrations in the medium. The scattering characteristics only depend on temperature, which may present an advantage when temperature is the sensing parameter of interest since there are no cross-sensitivity effects. However, Raman scattering in optical fibers has a much higher power threshold than Brillouin scattering. Therefore, Raman-OTDR is used for temperature distributed sensing [82] because thermally excited acoustic waves generate the spontaneous Raman scattering process rendering the scattering cross-section temperature-dependent. Distributed temperature sensing with Raman scattering has a temperature resolution of 0.5 °C with a measurement range up to 15 km at 1 m resolution (or up to 25 km at 1.5 m resolution) [27].

OTDR technology is used for distributed sensing and has significantly evolved in recent decades to the extent that nowadays there is accessible equipment providing a loss distribution map with a spatial resolution of a centimeter [83]. Examples of OTDR-distributed-sensing applications can be found in Section 3.2.

### 2.4. Summary

Table 1 compares the different types of optical fiber sensors according to the measurand employed for sensing, the fields where they are used, the parameters that they measure, and the possible network configurations that are applied in each type of sensor. It also includes the typical performance specifications of some of these sensors.

## 3. Optical Fiber Sensing Networks

In many applications, it is desirable or even imperative to measure strain and temperature at multiple locations at the same time. Wireless sensors emerged to solve some of the difficulties. However, the power supply required for continuous operation and the inability to scale in large scenarios present some of their associated disadvantages. These and other issues are overcome by Optical Fiber Sensing Networks (OFSNs), which offer the possibility to support a large number of sensors in a single optical fiber with long unamplified transmission ranges, high bandwidth, low power loss, and enhanced data privacy.

In addition to the single-point sensing, OFSNs can be distributed or multi-point.

### 3.1. Multiplexing Techniques

In applications where the goal is to simultaneously monitor data from several sensors in a network, it is challenging to process this information simultaneously. Multiplexing solves this problem by combining signals from several sensors into one signal transmitted over the same medium, which is the optical fiber.

Information can be multiplexed in several ways, depending mostly on the network topology (defines how sensors are connected in the network, more details can be found in Section 3.3) and on sensor addressing (used to distinguish information from each sensor in the network). This can be achieved considering different domains, such as the time, frequency, or wavelength. Ideally, there is a set of criteria that a multiplexing system should satisfy [84]: no restrictions regarding the type and properties of the sensors; scalability to increase the number of sensors in the network without penalizing its structure; ability to integrate sensors with high dynamic range; low interference (crosstalk) between sensors. Ultimately, multiplexing optical fiber sensors minimizes system costs since it allows for reductions in the optical sources, detectors, modulators, and other components necessary to support the sensing network [84].

The most common multiplexing techniques are space, time, frequency, wavelength, spatial-frequency, coherence or polarization. In this paper, we will focus on some of the most commonly used multiplexing methods for optical fiber sensors.

#### 3.1.1. Time Division Multiplexing

Time Division Multiplexing (TDM) is one of the most popular multiplexing techniques, where the measurements from each sensor are obtained considering the time of flight of light pulses [85]. In fiber arrays with FBGs, TDM takes advantage of the Bragg gratings which have a specific position along the length of the fiber. This allows it to launch short pulses and to anticipate the reflections from these pulses according to each sensor position along the fiber. Since each sensor is situated at a specific location, reflections caused by the optical signal are detected on the receiver with a delay determined by the distance between the optical source and the position of the Bragg grating. This method allows users to distinguish signals from different sensors placed in different positions along the length.

TDM is frequently used in the multiplexing of interferometric sensors and FBGs [85,86], where the combination of TDM and Wavelength Division Multiplexing (WDM) is also explored, allowing for the spectrum to be reused multiple times and thus increasing the number of sensors that can be addressed.

#### 3.1.2. Wavelength Division Multiplexing

WDM is used in FBGs along a fiber, where the number of supported sensors is limited by the reflective band of each FBG and the bandwidth of the light source. Since each sensing grating has a distinct wavelength, they do not cross each other, allowing users to multiplex many sensors along the same fiber.

An example of an implementation of WDM is provided in [87]. In this paper, WDM is applied to multiplex four FBG sensors, each with a distinct nominal wavelength ($\lambda_{1}$ = 1534 nm, $\lambda_{2}=$ 1542 nm, $\lambda_{3}=$ 1548 nm, $\lambda_{4}=$ 1557 nm) which permits strain sensing on each sensor. An interferometric interrogation approach is explored with a Mach–Zehnder interferometer. Strain causes a shift in the grating wavelength in FBG sensors, which gives rise to a change in phase that is detected at the interferometer output. This signal is then coupled into a bandpass WDM that separates the interferometer phase signal into discrete channels, which correspond to the wavelengths of the FBG sensors in the fiber. Finally, signals from each channel pass through a photodetector and a passive phase-shift carrier demodulator to retrieve the phase-encoded strain information. In conducted experiments, channel-to-channel crosstalk was residual, demonstrating the effectiveness of this multiplexing technique since the nominal wavelengths of each sensor do not cross each other [87].

Since it is used mostly to multiplex FBG sensors, the WDM multiplexing scheme is typically employed in temperature or strain sensing [86,87,88,89].

#### 3.1.3. Spatial Frequency Multiplexing

Spatial frequency multiplexing is a technique that combines signals in the spatial frequency domain. This is possible because each sensor has a specific spatial frequency that is distinct from the other sensors, and hence experiences low interference from other sensors.

An example of spatial frequency multiplexing is given in [90] where three sensors are multiplexed in the same channel of the interrogator. Multiplexing these sensors in series is possible because the spatial frequency is dependent on the width of the micro-fiber, and each sensor uses a micro-fiber with a distinct width. In conducted simulations, the spatial frequency multiplexing of these sensors achieved crosstalk as low as 0.2 rad/mm.

The spatial frequency multiplexing technique proposed in [91], explores a digital imaging processing technique to greatly reduce the number of fiber optic devices in the network and simplify the system architecture. In this system, several sensing fibers are connected to a cabled fiber bundle (coupler) that carries light to a Fourier transform lens, which allows an image to be sampled by a linear CCD array (converts an optical image into an analog signal). The image sampled by the CCD is then digitized and sent to a computer for processing. Conducted simulations showed that the phase information in an optical fiber sensor array can be encoded in fringe patterns and later retrieved by digital imaging processing techniques with a non-iterative phase-retrieval algorithm.

#### 3.1.4. Other Techniques

There are also other known multiplexing techniques, e.g., coherence [92], spatial, other frequency-based variants [84], and hybrid approaches. In the coherence multiplexing method, a short-coherence-length continuous optical source is used to encode information in the optical carrier components which have a certain mutual coherence value with respect to a reference optical carrier. This technique is mostly used in interferometric sensors as it takes advantage of the change in the relative phase between the two arms of the interferometer. Further details of this method can be found in [92]. Spatial multiplexing, described in [93], consists of using multiple spatial channels to transmit information. Data from each channel are transmitted via a dedicated optical fiber. Despite its simplicity, this technique requires a significant length of fiber necessary to multiplex a large number of sensors. However, as an advantage it is free from crosstalk between sensors and has a low noise level. This technique offers other advantages when combined with other multiplexing techniques, e.g., TDM [94] orWDM [95]. These are known as hybrid techniques. Other combinations of multiplexing techniques have also been used in the past, e.g., WDM + TDM [96], or spatial + TDM + WDM [95].

### 3.2. Distributed Sensing

The principle behind distributed sensing is the scattering of light that propagates in the fiber core, in particular, the backscattering to allow the propagation of the scattered light back to the detection unit. As previously explained in Section 2.3, optical time domain reflectometry is used in distributed sensing to determine the location of variations along the length of the fiber. A Raman-OTDR is used for distributed temperature measurements and a Brillouin-OTDR is used for distributed strain or temperature measurements. Sensing systems based on Brillouin and Raman scattering are used to detect the localized strain and temperature, allowing the monitoring of hundreds of kilometers along a structure with a single instrument, and with an accuracy of around 1 m [11,97].

**Figure 8:**
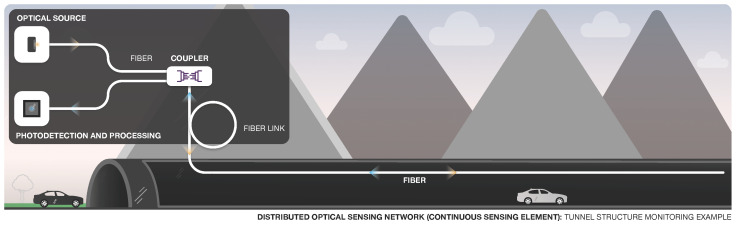
Distributed sensing based on continuous-sensing element.

The ability to measure temperatures and strains at thousands of points along a single fiber is particularly interesting for monitoring large structures [11,98] such as dams, tunnels, pipelines, bridges, and landslides, where it allows the detection and localization of movement, leakage, deflection, and seepage zones, with sensitivity and localization accuracy unattainable using conventional measurement techniques [6]. Figure 8 illustrates an application scenario for distributed sensing, where a continuous-sensing fiber element is used to monitor parameters such as strain or temperature in a tunnel.

In addition, OTDRs are widely used in distributed fiber networks as a diagnostic tool to monitor optical fiber communication links [79].

### 3.3. Multi-Point Sensing

Multi-point sensing detects variations only in the vicinity of localized sensors. Measurements performed in discrete points that can be located along a large area covered by an OFSN are considered multi-point sensing with multiplexed point sensors.

Multiplexing involves the concepts of network topology, sensor addressing, and sensor interrogation. The first is related to the sensors’ arrangement in a network, which may have consequences in terms of power budget and sensor crosstalk. The second involves the study of processes and techniques that permit the addressing of a particular sensor from the emission and detection block, taking into consideration that typically all sensors are related to time, wavelength, coherence, frequency, or spatial addressing. The third is associated with the process to read the status of a specific sensor and obtaining measurand information.

In multi-point sensing, there are multiple types of topologies: serial, parallel, and ladder [99,100]. The serial topology consists of an optical source, a modulator, a sensor array, and to recover the signal, a demodulator, and finally, the optical detector. Sensors can be reflective or transmissive, which means that different configurations influence the way the signal is redirected to the detector array. A reflective sensor reflects the light in direction of the detector and a transmissive sensor redirects the signal to the detector.

The parallel topology is the simplest arrangement and consists of an optical source (which can be more than one source) that is coupled into the fiber network, and the power is distributed via a multiple-port directional coupler into a set of parallel downleads. The signal travels through each downlead that contains a sensor and is then redirected to the detector array.

**Figure 9:**
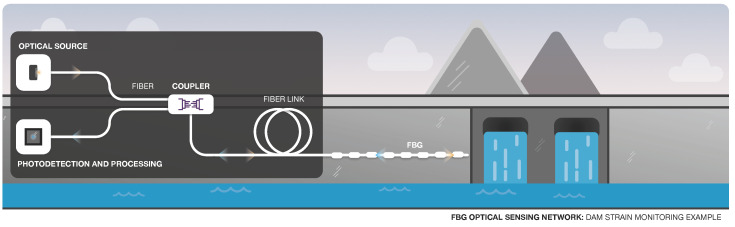
FBG Interrogator in a Structure Strain Sensing Application.

FBG networks (Figure 9) are one of the most well-known examples of multi-point sensing. FBGs are widely used owing to their many advantages already described before, such as multiplexing capability and wavelength-encoded information, and eliminating power variations [86,101,102]. FBGs can measure the strain and temperature separately [11].

These devices are inherently self-referenced since information is encoded in the resonant wavelength of the structure, which is an absolute parameter and can be easily multiplexed. This is particularly important in the context of multi-point sensing. The conversion of the Bragg wavelength value and variations into an electrical signal to obtain the information is designated by FBG interrogation.

In order to use sensors over very large distances, optical amplifiers can be employed along the fiber network [103]. Figure 9 illustrates a simple example, where an FBG sensing network is applied to monitor the strain in a dam.

In Portugal, this technology was adopted for real-time monitoring of structures and the environment [18,54]. In 2004, an OFSN was implemented in the bridge Luis I in Porto. More than 120 sensing points were addressed with FBGs located in specially designed optical fiber cables spanning several kilometers.

**Figure 10:**
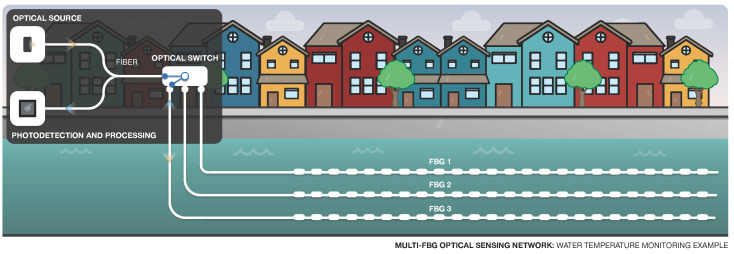
Representation of a Multi-FBG sensing system for measurement of water temperature.

In 2002, a sensing network based on FBGs was implemented at Ria de Aveiro (Portugal) to monitor the distribution of water temperature along the 12 km extension [18]. Figure 10 illustrates a similar application scenario, where an optical switch allows for the interrogation of different FBGs.

FBGs are also implemented in many other domains, such as aeronautics, land and ocean transportation, oil and gas exploration, steel corrosion monitoring [104], as well as environmental monitoring.

Some areas of application where optical fiber distributed sensing is commonly used can also be addressed with FBGs. New developments in FBG interrogation allows for the reading of a large number of closely distributed, equal-wavelength, low-reflectivity FBGs with high resolution, demonstrating a very effective solution based on this technology [18].

Table 2 shows a comparison between multi-point and distributed sensing using FBG or OTDR for sensing temperature and strain, which represents the most common use of these optical fiber sensors.

We compare between commercially available sensors to understand the fundamental differences and the applications where they are better suited. In this case, we list the specifications of distinct FBG sensors for temperature and strain sensing and compare them with a Brillouin-OTDR that performs simultaneous sensing of these parameters. The main differences exist within the sensing itself, where multi-point sensing performs sensing in multiple discrete points along the fiber, and distributed sensing allows the obtainment of measurements over a continuous length of fiber with a spatial resolution of 1 m for a fiber length up to 10 km. In many cases, the distance between Bragg gratings can be customized to match the user’s needs. Although there is a possibility for simultaneous sensing using FBGs [105], most available commercial FBGs are developed for either temperature or strain sensing bearing different specifications in each case. This is a clear advantage of Brillouin-OTDR since it can simultaneously measure temperature and strain. Measuring the Brillouin scattering enables sensing over longer distances with a distance range that spans from 100 m up to 200 km, while the interrogation methods in FBGs allow for a length of fiber up to 20 km without amplification. Regarding the sensing capabilities, Brillouin-OTDR bears larger temperature and strain ranges than FBGs. The hardware necessary to extract measurand information is different in multi-point sensing and distributed sensing considering FBGs and Brillouin-OTDR, where the first depends on interrogators and the second requires a Brillouin reflectometer.

### 3.4. OFSN Applications

As previously discussed, since the optical fiber is made of non-metal material, it is more resistant to environmental factors and can be used in severe conditions such as high/low temperature and humidity. In addition, it is immune to electric and electromagnetic interference as well as signal errors in the transmitting process. Therefore, OFSNs have been used in several fields for monitoring different parameters. The dual functionality of the fiber (simultaneously sensing element and communication channel) with low limitations (distance versus bandwidth) enables the implementation of an extensive sensor network. Hybrid approaches based on optical fiber sensing combined with out-body wireless communication are also interesting in this domain [18].

This section presents a summary of applications of optical fiber sensor networks in different fields, specifically considering multi-point and distributed sensing. In several applications, a combination of distributed and multi-point sensing can be used, e.g., in healthcare sensing systems [18]. Examples of single-point sensing were already provided for each type of optical fiber sensor in Section 2.

The lifespan of the infrastructure is long, ranging from several decades to over one hundred years. Depending on the type of structure, a range of parameters such as strain, temperature, corrosion and thickness reduction, leakage acoustics, and pressure may be important to quantify. Despite the existence of several methods to detect damage in civil infrastructure [110], most of them suffer from various disadvantages such as lack of portability, susceptibility to electromagnetic interference, and lack of capability for continuous and remote monitoring over large distances. In 1989, optical fiber sensors were introduced as concrete structural monitors [12] and became a very important element in civil infrastructures, such as bridges or pipelines [11]. Subsequently, many research groups started to implement optical fiber sensors in various structures [9,12,18].

Optical fiber strain sensors have shown to be the best option for long-term health monitoring of concrete bridges due to their small size, light weight, immunity to electromagnetic disturbance, resistance to harsh environments, ability to be embedded internally, and multiplexing capabilities. These sensors have been used for measuring strain in large concrete and steel structures [15,54,111], for health monitoring in composite structures [112] and railways [113], for monitoring structural fires [114], and for strain/displacement monitoring of geotechnical structures (dams, slopes, tunnels, or excavation engineering) [115]. In addition, OFSNs are capable of long-distance transmissions, hence they are better suited for remote strain monitoring than any other strain sensing technique [12].

FBGs with multi-point sensing network configuration were used to create seismic maps of the sea floor, and these maps are used today for monitoring oil and gas reservoirs [116]. This is achieved by analyzing the propagation of seismic waves induced by controlled explosions. Interferometric optical fiber sensing systems were used to sense the seismic waves and may require over 30,000 sensors, using a specific combination of time and wavelength multiplexing [18].

OFSNs provide sensing solutions for almost all application types and in inflammable, radioactive, or chemically corrosive environments, owing to the intrinsic characteristics of the optical fiber [18,117,118]. In the following, a few additional applications are listed:

- Electric and magnetic fields: A summarized overview of fiber optic sensors for measuring electric and magnetic fields was proposed in [119], which are used in single point sensing configuration for localized measurements.
- Localization of heat sources: Chirped FBGs have been used for localization of heat sources and shock wave detection, among other applications [120].
- Battery management: A study to assess the possibility of integrating fiber optic sensors to monitor the battery health (temperature, strain, and humidity) was carried out in [121].
- Monitoring aerial vehicle structural health: In aviation, FBG networks are explored to monitor an aircraft’s structural health [47,122] and to monitor a spacecraft’s structural health (temperature and strain) [123].
- Healthcare: Heart rate and respiratory rate sensors based on FBGs perform multi-point sensing in [62]. A review of fiber optic sensors for sub-centimeter spatially resolved measurements [124] presented applications of these sensors in many areas, such as thermotherapy, catheterization for diagnostic purposes through gastroscopy, urology diagnostics, and smart textiles.

## 4. Wireless Sensor Networks

With the development and proliferation of Micro-Electro-Mechanical Systems (MEMS) technology, which enables the development of smart sensors, WSNs have attracted worldwide attention [125,126,127,128]. These sensors can be designed to measure different parameters (e.g., mechanical, thermal, biological, chemical or magnetic) and can be small and low power. Integrating wireless communications capabilities with these sensors allows for the creation of low-cost nodes that are able to acquire and transmit data. Due to these features, WSNSs provide a flexible and efficient approach for multi-point sensing, allowing large-scale real-time monitoring and data collection and at a lower cost.

**Figure 11:**
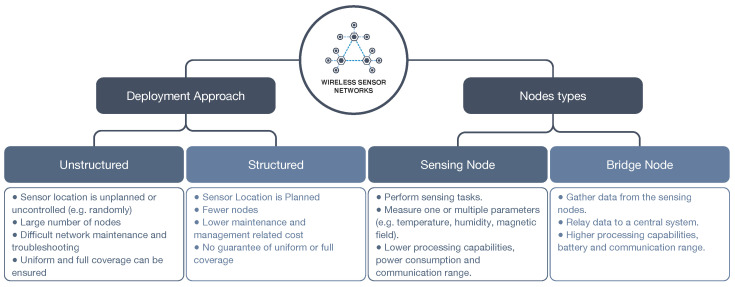
Wireless Sensor Networks: Deployment and Node Types.

In a WSN, sensor nodes work together to monitor several parameters about the environment, usually without an infrastructure. Considering the deployment approach, a WSN can be classified as unstructured or structured [125] (Figure 11). An unstructured WSN contains a large number of sensor nodes that can be deployed randomly (in an ad hoc manner), increasing the complexity and costs related to network maintenance (e.g., node troubleshooting). In addition, since the locations of the nodes are not specified, uniform or full coverage of an area cannot be guaranteed. In a structured WSN, the sensor nodes’ locations are planned and plotted. Therefore, uniform and full coverage can be ensured and fewer nodes can be deployed, resulting in lower costs for network maintenance. However, the structured deployment can be complex in certain applications and lead to more effort and costs in the deployment stage.

Two different types of nodes can be deployed in a WSN: sensing nodes and bridge nodes (or gateway nodes) [125] (Figure 11). The sensing nodes perform the measurements or data acquisition and relay the data to nearby bridge nodes, while the bridge nodes gather data from multiple sensing nodes and relay the data to a base station or a central system. Bridge nodes have higher processing capabilities and power consumption, but this approach allows for a reduction in processing requirements and power consumption in the sensing nodes. In addition, different radio communications can be used between the sensing and bridge nodes and between the bridge nodes and the base stations also reducing the cost of the overall deployment.

Sensor nodes are normally powered by batteries, and despite the low power consumption, the requirement of batteries still presents a limitation. In some applications, batteries can be charged by solar energy.

WSNs can be deployed in different environments, which means we can have terrestrial, underground, underwater, multi-media or mobile WSNs. For more details regarding the types of WSNs and their applications, we refer the reader to [125].

### Integration with Optical Fiber Sensors

As mentioned before, MEMS sensors have some limitations in terms of measurement capabilities, when compared to optical fiber sensors, due to the limited types of sensing mechanisms built into the MEMS sensor design. On the other hand, optical fiber sensor systems are often used alone in pre-defined positions, and therefore extensive lengths of optical fiber cable are necessary for connecting all the sensors as an optical fiber network. Additionally, in many circumstances dealing with this cabling can be a problem.

Despite the distinct advantages of WSNs and optical fiber sensors, and the constant developments in both areas, the integration of these two technologies is not a common occurrence. The reason behind this is possibly related to the complexity of optical fiber sensors in comparison to other sensors, and the relatively low cost and low power consumption of other solutions. However, these “hybrid” sensing networks may be very important in applications where harnessing the advantages of both optical fiber sensors and WSNs is crucial [129].

There is some research focused on the integration of WSNs with optical fiber sensors [22,130,131], aiming to obtain the best of both worlds, namely the advantages related to the optical fiber sensors and the means of communication provided by WSNs.

**Figure 12:**
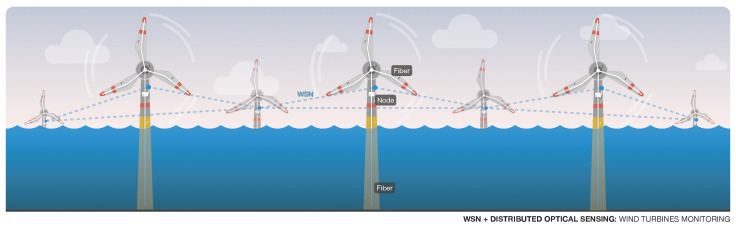
Representation of optical sensing and WSN integration.

Figure 12 represents a possible application of a solution that integrates optical sensing with a WSN. In the depicted case, a distributed optical sensing network is used to monitor each wind turbine in an offshore wind farm. The parameters that can be monitored include underwater structure bend, corrosion, water temperature, salinity, and so forth [132]. Moreover, optical fibers can be used to monitor blade parameters [133], such as the bend or strain, that influence the performance of the wind turbine. In addition to the immunity to electromagnetic effects, another advantage is that optical fibers are ideal for performing this type of monitoring because it is flexible and light, and can even be integrated in to the blade material [134].

All of the measurements can then be sent using a WSN, where the nodes installed in each turbine work together to relay the data to a central monitoring server. This approach is especially useful in deployments that lack a dedicated communication link. With regard to agriculture, optical sensors can also be used to measure several parameters related to the environment, field and crops [135], and the integration with WSN is also an advantage to wirelessly gather the data from multiple sensing nodes.

In [22,136], a design is presented for a wireless mobile platform to locate and gather data from different types of optical fiber sensors. This can be used in applications such as monitoring in remote and harsh environments and tracking, exploiting fully the combined advantages offered both by the mobile WSN and the advanced optical fiber sensing technologies. In the presented platform, an optical fiber sensor module and a smart mobile WSN module are used, showing important advantages in mobile sensing. The authors designed a temperature sensor based on FBGs and an intrinsic pH optical fiber sensor, and then integrated them successfully into a WSN platform.

More recently, Su-Ping et al. [130] proposed a hybrid system that was deployed in a borehole for real-time remote capture of subsurface deformation. The main architecture of this system is composed of three modules, the sensing module, the transmission module, and the cloud module. The sensing module comprises various sensor nodes based on FBGs which are used for measuring strain in the borehole. The data collected by the sensing module are wirelessly sent by the transmission module (based on 4G/5G cellular networks) to the cloud module. Upon receiving the data, the cloud module processes and analyses the data to output the vertical strain information, which allows the results to be compared with historical data and to send alerts when certain thresholds are exceeded.

The work described in [131], proposes a real-time system for underground mining monitoring based on optical sensors and WSN. First, it discusses the importance of sensor node deployment in underground mines. Then, it introduces an architecture that may detect and localize sources of damage such as strain vibration, temperature, and humidity change, using FBGs, Brillouin and Raman OTDR, and interferometers. The successful creation of this type of “hybrid” solution can thus be expected to make an important impact in several areas, where either conventional optical sensor designs or WSNs alone cannot meet the systems’ requirements [22].

The industry benefits from the integration of WSNs with optical fiber sensors for monitoring the aging process of industrial assets [137]. This approach explores low-cost photonic sensors for monitoring the aging process of coolant fluid. Each sensor is then packaged into a WSN node, which can be deployed in remote places in an industrial environment. This results in a dynamic WSN which is adaptable for continuous monitoring and management of industrial assets. Consequently, this approach can be applied in a variety of industrial and environmental monitoring applications.

There is a highly prominent recent research topic for uniting WSN with optical sensing networks, which are smartphone-based interrogator systems [138,139]. In these systems, the smartphone is both the optical source (LED camera) and detector using the camera to collect the optical signal, and then it performs interrogation to obtain information about the measurand.

In [138], a fiber optic temperature-sensing system integrated with a smartphone platform was proposed. The sensing system is composed of a fiber-based surface plasmon resonance sensor which uses the LED of the smartphone as a light source from one end to emit the input signals. Output signals are recorded and processed by the smartphone camera on the other end of the fiber. Experimental results demonstrated that this temperature sensor can achieve a measurement resolution of 0.83 °C with an operating range between 30 and 70 °C.

More recently, Markvart et al. introduced the smartphone-based interrogation of an FBG sensor [139], consisting of a smartphone and low-cost off-the-shelf available components. The LED flashlight acted as the light source and was coupled into a chirped FBG, inscribed with multimode fiber. To perform the interrogation, the other end of the fiber was coupled into the smartphone camera. A piece of a DVD disk was placed between the camera lens and the end of the fiber to act as a low-cost diffraction grating. Validation was performed with a commercial spectrometer (used as a reference) to compare its performance against the smartphone interrogation for strain sensing. The results demonstrated that the characteristics of the smartphone-based spectrometer were similar to those of the commercially available spectrometer, achieving picometer-level Bragg wavelength and micro-strain level measurement resolution.

Although it is clear that these approaches produce less accurate measurements than conventional interrogation systems, these advances will pave the way for low-cost portable sensing systems that combine the advantages offered by optical sensing and smartphones. These solutions bear potential applications in healthcare, environmental monitoring, or other fields.

## 5. Conclusions and Future Prospect

This paper presented a literature review and discussion regarding the principles, applications, and characteristics of optical sensing that make this technology unique. The paper started with a description of the different types of optical fiber sensors, their characteristics and operating principles, followed by a discussion about Optical Fiber Sensing Networks (OFSNs). The characteristics of multi-point and distributed sensing were presented. The high potential of OFSNs, particularly the ability to perform simultaneous measurements over a long and continuous length of optical fiber, and the ability to measure different parameters using the same sensing element are valuable features for monitoring large structures or environments where other sensing technologies are challenging or impossible to deploy. Therefore, the presented literature review clearly shows that OFSNs can be applied in different areas, such as environmental monitoring, civil and mechanical engineering, and their versatility was highlighted via concrete examples that were presented and evaluated. The characteristics of two commercial solutions for temperature and strain sensing were compared.

In this work, we identified several areas of potential future developments, namely optical fiber sensors and sensing networks, and the integration of optical fiber sensing with WSNs.

Optical fiber sensing technology is contributing toward the development of new sensors and approaches for sensing numerous parameters, e.g., temperature, strain, water levels, presence of chemicals (DNA, pH, ammonia, etc.). Despite this, there are still some engineering challenges to overcome before achieving good stability, high accuracy, compactness, and reasonable costs which represent the conditions necessary for large-scale commercial application of the interrogation systems. Recent papers have shown that this is an active field of investigation with potential in terms of research and applications in various fields (healthcare, oil industry, civil engineering, etc.).

Finally, the integration of optical sensing with WSNs, a topic that is infrequently discussed, was analyzed regarding the potential benefits of employing a hybrid architecture that allows the application of optical sensing and takes advantage of the mentioned benefits, in addition to harnessing the benefits of wireless networks. This integrated approach is relatively recent but can significantly evolve and attract the attention of researchers in the future, especially considering the fast development of the Internet of Things (IoT), the 5G communication networks, as well as innovations in optical fiber sensor technology. The use of smart devices is increasing exponentially owing to their practicality, portability, low cost and connectivity, which make them suitable as nodes for WSNs, while also contributing toward its development and future applications. Furthermore, the computing resources and connectivity offered by smartphones combined with the latest developments in smartphone-based interrogation can bring new developments on the integration of optical fiber sensing networks with WSNs.

## Figures and Tables

**Table 1:** Comparison between fiber optic sensors.

Sensor	Measurand	Field(s)	Sensing Application(s)	Network Config	Performance [27]
Fiber Bragg Gratings	Wavelength shift	Engineering, Physics, Cryogenics	Temperature, pressure, strain, liquid level, displacement, salinity.	Single/Multi-point sensing	Strain res. < 0.5 μϵ Long-term accuracy < 1% Temp. res. 1 °C
Interferometers	Phase-shift in light	Navigation, Engineering, Networks	Gyroscope (inertial navigation, surveying, defense), hydrophone (refractive index sensor, pressure monitoring of structural components), and optical switching.	Single/Multi-point sensing	N.A.
Rayleigh-OTDR	Rayleigh scattering	Engineering, Physics, Cryogenics	Distributed acoustic sensing.	Distributed sensing	Freq. range 1 mHz to 100 kHz Spatial res. 1 m Length up to 50 km
Raman-OTDR	Raman scattering	Engineering, Physics, Cryogenics	Long-distance and real-time monitoring, distributed temperature sensing.	Distributed sensing	Temp. res. 0.5 °C Meas. range up to 15 km w/ 1 m spatial resolution Meas. range up to 25 km w/ 1.5 m resolution
Brillouin-OTDR	Brillouin scattering	Engineering, Physics, Cryogenics	Long-distance and real-time monitoring, distributed temperature, and strain sensing.	Distributed sensing	Typical strain res. 20 μϵ but can achieve up to 0.1 μϵ Meas. range up to 10 km w/1 m spatial resolution, but supports larger ranges w/reduced accuracy Temp. res. 0.5 °C

**Table 2:** Comparison between multi-point sensing and distributed sensing using FBGs or a Brillouin-OTDR.

	**Multi-Point Sensing** **FBG (Temp. [106], Strain [107])**	**Distributed Sensing** **Brillouin-OTDR [108]**
Spatial Accuracy	Physical parameters measured at multiple discrete points	Physical parameters measured continuously throughout the length of the fiber
Spatial Resolution	Depends on distance between Bragg gratings (can be customized)	1 m (up to 10 km); 5 m (up to 60 km)
Simultaneous sensing	Distinct FBG sensors must be used for sensing temperature or strain	Simultaneously measures temperature and strain
Dist. Range (max. length)	20 km (without amplification) [109]	100 m to 200 km
Temp. Range (°C) Resolution (°C) Accuracy (°C) Temp. Repeatability (°C)	Up to [−40, +250] 0.1 From 0.1 to 1.0 N.A.	[−200, 700] N.A. N.A. 0.8
Strain Range (μϵ) Resolution % Accuracy % Strain Repeatability (μϵ)	Up to [−3000, 3000] From 0.1 to 2.0 From 0.1 to 1.0 N.A.	[−30,000, 40,000] N.A. N.A. 17
Extract measurand info.	Demodulators/interrogators	Brillouin Reflectometer

## Data Availability

Not applicable.

## Abbreviations

The following abbreviations are used in this manuscript:

FOS	Fiber Optic Sensor
FBG	Fiber Bragg Grating
IoT	Internet of Things
MEMS	Micro-Electro-Mechanical Systems
OFSN	Optical Fiber Sensing Network
OTDR	Optical Time Domain Reflectometer
PCF	Photonic Crystal Fiber
TDM	Time Division Multiplexing
WDM	Wavelength Division Multiplexing
WSN	Wireless Sensor Network
